# Perspectives of Canadian privacy regulators on anonymization practices and anonymized information: a qualitative study

**DOI:** 10.1093/idpl/ipae017

**Published:** 2024-12-18

**Authors:** Khaled El Emam, Anita Fineberg, Elizabeth Jonker, Lisa Pilgram

**Affiliations:** Khaled El Emam, School of Epidemiology and Public Health, University of Ottawa, Ottawa, Ontario, Canada; CHEO Research Institute, Ottawa, Ontario, Canada; Anita Fineberg, Chang School of Continuing Education, Toronto Metropolitan University, Toronto, Ontario, Canada; Elizabeth Jonker, CHEO Research Institute, Ottawa, Ontario, Canada; Lisa Pilgram, School of Epidemiology and Public Health, University of Ottawa, Ottawa, ON, Canada; CHEO Research Institute, Ottawa, Ontario, Canada; Department of Nephrology and Medical Intensive Care, Charité—Universitaetsmedizin Berlin, Berlin, Germany

Key Points

- There is a lack of precision and consistency across Canada in regulating anonymization practices, in defining anonymized information, and there is no generally accepted national guidance on how to anonymize data. This has resulted in an environment that impedes the ability to have national technical and policy solutions for anonymization, to share anonymized data across jurisdictions, and to provide certainty for organizations that operate across jurisdictions.
- To understand the perspectives of privacy regulators on anonymization practices and how to regulate anonymized data, we performed a qualitative interview study with 93 per cent of privacy regulators in Canada.
- Despite heterogeneity in perspectives, the main findings where there is consensus are: specific consent is not required for the process of anonymization, although transparency is important; proper anonymization should be ensured in a proactive way through, for example, Codes of Practice; and anonymized data should not be free of oversight. But there is variation in views on the degree of oversight needed, as well as on the need for and approaches to restrictions on the purpose for processing anonymized data. Because some fundamental anonymization issues are open to interpretation by an incumbent regulator, this increases uncertainty and results in inconsistencies across the country.
- Our recommendations are to implement a national Code of Practice to ensure properly anonymized data (with possible sector-specific adaptations), incentivize anonymization through reduced regulatory obligations on process and output, explicitly address key anonymization parameters in legislation and regulation, and incorporate ethical considerations into anonymization practices.

## Introduction

Training artificial intelligence and machine learning (AIML) models requires large datasets.^1^ However, access to data for AIML projects has been problematic in practice. Both the US Government Accountability Office^2^ and the McKinsey Global Institute^3^ note that difficulties in accessing data for building and testing AIML models are an impediment to their adoption more broadly. A Deloitte analysis concluded that data access issues are ranked in the top three challenges faced by companies when implementing artificial intelligence (AI).^4^

Getting access to data is a challenge due to privacy concerns. One survey highlighted the privacy concerns of companies adopting machine learning models, with more than half of companies experienced with AIML checking for privacy issues.^5^ As a further example, in the case of health data, privacy concerns by patients and regulators have acted as a barrier to the sharing of health data.^6^ A recent review of health data infrastructure in Canada concluded that (mis)interpretations of privacy laws and a general ‘privacy chill’ incentivize risk-averse behaviour among data custodians, stifling data access.^7^

One approach to enable data sharing and data access is to anonymize the data. However, the regulatory definition of concepts, such as anonymization, identifiability, and personal information, as well as their translation into technical standards varies across national, sub-national, and local levels, making it difficult for organizations to determine how to assess re-identification risk and anonymize information consistently.

Canada exemplifies the complex dynamics of privacy regulatory governance, reflecting the division of powers between the federal and provincial governments. Federally regulated governmental institutions and businesses are regulated by the Privacy Act and the Personal Information Protection and Electronic Documents Act (PIPEDA). PIPEDA also applies to provincially regulated businesses apart from Alberta, British Columbia, and Quebec. In June of 2022, the federal government introduced the *Digital Charter Implementation Act, 2022*^8^ (known as Bill C-27) which, among other matters, would substantially amend PIPEDA. Outside the private sector, the landscape is even more fragmented with some provinces having separate health or employment-related privacy regulations. In addition, there are numerous privacy requirements that are within (non-privacy) sector-specific laws.

The Supplementary Materials summarize the quite different ways in which the concepts of personal information, anonymization, and identifiability have been defined in these statutes for the private sector (Supplementary Table S1), health sector (Supplementary Table S2), and public sector (Supplementary Table S3). Recent opinions from the Ontario regulator^9^ and the federal privacy commissioner^10^ provide some specific examples of what are deemed to be acceptable anonymization practices, and current Canadian guidelines consist of the Ontario guidance from 2016^11^ and the recent Quebec regulations.^12^ Yet, these are not aligned with each other on key parameters and are imprecise on important criteria. For example, in Ontario’s Personal Health Information Protection Act (PHIPA), anonymization is a permitted use, but not under PIPEDA. In Quebec’s private sector law anonymization must be performed according to generally accepted best practices, but not under Ontario’s PHIPA,^13^ and the federal privacy commissioner has argued against incorporating ‘generally accepted best practices’ in the definition of anonymization in Bill C-27.^14^ Furthermore, the Quebec regulations respecting the anonymization of personal information^15^ define three anonymization criteria modelled after an opinion from the European Article 29 Working Party on Anonymization Techniques,^16^ but these do not exist in other Canadian regulations: correlation, individualization, and inference. Both the current state of Canadian privacy laws and the recent amendments to PIPEDA proposed in federal Bill C-27, do not provide the country’s privacy regulators with *precise and consistent* direction for interpreting and applying the legislative provisions regarding anonymization.

This means that the regulation of anonymization practices and anonymized information can vary by Canadian jurisdiction, which impedes the ability to: have national technical and policy solutions for anonymization, share anonymized data across these jurisdictions, and provide certainty for organizations that need to operate across jurisdictions or nationally. Such challenges are not unique to Canada and have been documented in the general literature and in non-Canadian jurisdictions.^17^

To understand this regulatory landscape and how Canadian privacy regulators (commissioners and ombudspersons) currently regulate anonymization practices and anonymized data, and the changes they would like to see, we conducted a qualitative interview study with them in the first quarter of 2022. These interviews aimed to collect and organize information on anonymization in general and were not explicitly tailored towards a particular sector. Based on the findings, we developed pan-Canadian anonymization recommendations that can reduce friction in using and disclosing data for secondary purposes, such as training AIML models.

## Methods

For the purposes of this article, we will use the term ‘anonymized’ and ‘de-identified’, and their verbs, interchangeably to mean information for which there is no serious possibility in the circumstances that it could be associated with an identifiable individual.^18^ The terms ‘pseudonymisation’ and ‘pseudonymous’ will be used when directly identifying information, such as names, telephone numbers, or social security numbers, are replaced by a pseudonym. Data that have been pseudonymised are still considered personal information.^19^

We performed a qualitative study to explore regulators’ perspectives on contemporary anonymization methods to facilitate access to data for secondary purposes. The coronavirus disease 2019 (COVID-19) pandemic highlighted the need for sharing multiple types of anonymized data.^20^ The study was conducted at the tail end of the COVID-19 pandemic, which ensured that the high demand for data was salient and the related privacy concerns were also top-of-mind.

While examples were primarily drawn from the health and private sector, the interviews were not tailored towards a specific sector. The interviews were meant to capture overarching aspects.

### Study participants

Canada has 10 provinces and 3 territories all of which have their own privacy commissioner or ombudsperson responsible for the respective privacy legislation. The Office of the Privacy Commissioner of Canada is the federal privacy regulator. All of the privacy regulators in Canada were invited directly or indirectly through their offices to participate in the interviews.

Some offices of federal, provincial, and territorial privacy commissioners or ombudspersons within Canada sent 2 interviewees to the interviews; therefore, in total, there were 16 participants in 13 interviews. One privacy regulator declined to participate, and therefore the coverage was 93 per cent (13/14) of all privacy regulators.

An important question in a qualitative study is whether it represents a sufficient number of participants. Common considerations in choosing the number focus on reaching saturation on the part of the interviewees and on the limit of reasonable encounters on the part of the interviewer.^21^ Saturation is the point at which there is nothing new to be uncovered on the topic.^22^ Once saturation is reached, further interviews are no longer necessary or particularly useful.^23^ A limit of encounters is given by the researcher’s own ability to recall, process, and understand the interviews.^24^ As Gaskell points out, ‘the interviewer must be able to bring to mind the emotional tone of the respondent and to recall why they asked a particular question’.^25^ Therefore, there is a limit to the number of encounters that the researcher will be able to recall in detail. For individual interviews, Gaskell suggests that this limit would be between 15 and 25 interviews.^26^

Given that ours is a finite population of at most 14 interviews, saturation probably occurred prior to complete coverage (ie, 14 interviews). With only one potential participant missing, we have very likely achieved it during our 13 interviews, without reaching the interviewer’s limitation of encounters. This is in line with the interviewer’s observations. The interviewer had a sense of participants’ potential viewpoints before commencing the interviews, as a result of the environmental and legal reviews completed in preparation for the interviews, and confirmed the sense of saturation.

### Study procedure

The interviews were conducted by videoconference and followed an interview guide developed by the researchers. The participants received background information including the interview questions ahead of the interview, and each interview started with general definitions of terms.

While the initial focus of the study was on synthetic data generation (SDG) as a privacy-enhancing technology (PET), it became clear at the very outset that the technology used was not very relevant from the regulators’ perspective, and the main issue was the anonymization method and regulating the anonymized data itself.

The interview questions therefore pertained to the following aspects:

- Does the privacy regulator have experience with anonymization and the use of PETs in their jurisdiction, either through investigations, submitted privacy impact assessments (PIAs), or consultations?
- Should the process of generating anonymized data require explicit data subject consent, and should it be regulated or overseen in other ways?
- Should the anonymized data be regulated or overseen?
- Should AIML models be regulated if they were trained using personal information or anonymized information?

The actual interview questions were probing the four aspects above in different ways. This helped to create a more comprehensive picture to generate recommendations on how to regulate anonymization practices and anonymized data.

The duration of each interview was between 45 and 60 minutes. The interviews were audio recorded and transcribed verbatim.

We used an approach informed by grounded theory^27^ to analyse the interview data. The objective of the analysis was to understand participants’ perspectives on the risks and benefits of using anonymization to facilitate access to data for secondary purposes. Data transcription and analysis were carried out in parallel to the interviews and continued until a set of stable themes developed. Following each interview, the interviewer conducted a cursory analysis of the audio recordings and field notes to determine if changes to the interview guide were required in preparation for the next interview. Questions may be added, removed, or reworded as required. Using the constant comparison method,^28^ we developed a coding scheme that embraced the themes presented in the data. Research team members experienced in qualitative research methods coded the transcripts using an inductive process. Once the members completed their independent coding, they compared their coding and discussed it to come to an agreement. Throughout the process, codes could be modified, merged, or eliminated as required to increase the accuracy of the analysis.

## Results

The results are grouped into the unique themes that emerged from the interviews and, to the extent possible, grouped to match the original questions. Where relevant, anonymous quotes from the interviewees are provided to re-enforce or explain a particular point of view.

### Experiences with anonymization

The interviewees had experience with complaints and investigations of anonymization practices and anonymized data. The anonymization methods used include traditional approaches, such as generalisation and suppression. There was limited experience with contemporary methods, such as SDG. One such experience was where SDG did come up in a complaint against Facebook and the use of synthetic data for software testing,^29^ and another was an ongoing project in Alberta on SDG that included consultations with the privacy commissioner.^30^ The vast majority, however, stated that they did not yet have any use case or internal discussions on SDG. Consequently, the remainder of the results focused on anonymization in general and remain technology agnostic.

### Regulating the process of anonymization

#### Obtaining consent for anonymization

The creation of anonymized data from real data is considered a (further) processing of the original personally identifiable data. On this particular point, there was overall agreement. Our questions further asked whether such processing of personal information requires or should require the consent of the data subjects. The consent of the data subjects would be obtained at the initial point of data collection, or data subjects would be asked for consent again when the data custodian decides to anonymize datasets.

There were four perspectives on the need to obtain consent for the act of anonymization under the current legislation:

1. The legislation makes it clear that the creation of non-identifiable datasets is a permitted use (as is the case under Ontario’s PHIPA^31^), and therefore no additional consent is required.
2. A strong case can be made that the application of anonymization, which further protects the rights of the data subjects by generating non-identifiable data, should be encouraged, and the regulator would be satisfied with that argument as long as the anonymization was appropriately executed (ie, followed best contemporary practices). In such a case, consent to anonymize data is not required.
3. If the purposes for which the anonymized data will be used or disclosed are consistent with the initial consent for which the personally identifiable dataset was collected, no consent is required. Whether a purpose is consistent or not needs to be interpreted on a case-by-case basis.
4. Explicit consent is required to create anonymized datasets.

The interviewees’ opinions spanned the four perspectives. However, the majority of respondents were of the opinion in 1 or 2. Therefore, there is a consensus that specific consent is not required for properly applied anonymization; however, there was not unanimity.

A number of conditions for not requiring consent were also stipulated by some interviewees:

- Only anonymization carried out internally or by health authorities would not require consent, while confidence in anonymization by third parties depends on further measures, such as data-sharing agreements.
- The implementation of further suitable controls for the anonymized data (see below).

#### Implementation of good practices

One of the concerns raised by the interviewees is that many public and private sector organisations have claimed to implement anonymization methods. But in practice, it was difficult to ensure that they followed good practices for anonymization. There have been examples of organizations just removing the names of data subjects and claiming that the data are anonymized. However, removing the names of individuals is one step in pseudonymization and may produce pseudonymous data, but it is very unlikely to result in datasets that meet contemporary definitions of anonymized data.

The interviewees would find out that poor anonymization practices were implemented only when there is a breach or a complaint. At that point, it is too late to have an impact on these practices, and there may already be damage or harm to the data subjects through privacy violations. Therefore, the regulators generally agreed that a more proactive process would be desirable. They noted that, ideally, they would provide organizations with guidance on how to perform proper anonymization as a way to support the implementation of good practices and also to support the regulators in evaluating claims. Most regulators further felt that something more verifiable and enforceable than just guidelines should be required to address the caveat of ‘if implemented properly’ that often accompanies the implementation of PETs in general.

*So, I think that some of the control probably should be […] how to do it properly so that there’s consistent expectations […].*

*[…] assuming that they did a good job of de-identifying the data, and of course […] that often is the key question here. Everybody knows what standards we’re trying to meet. It’s whether they’re actually met in a given case or not, that’s often the real issue.*

Consequently, some of the interviewees were concerned about how to ensure that these good practices are actually implemented and expressed further concern about self-regulation by industry.

One approach that was discussed was Codes of Practice. In the interviews, the term Code of Practice was used for a set of guidelines or rules that outline the standards and procedures that are implemented within a framework with control mechanisms and consequences for non-compliance. The following advantages were mentioned:

- They would be explicitly approved by a public body and therefore would have a stronger standing if an organisation does implement them.
- Codes of Practice would typically have a certification or audit mechanism to ensure that the practices are actually followed.
- They would typically include consequences for non-compliance.

Another similar proactive approach that was proposed was to apply recognised standards with certification bodies or companies that can audit against these standards.

*[…] one of the tools that could help in bringing the high general legal standard [downwards], would be codes of practice approved by the public body […].*

*I’m going to be cautious around use of the word ‘need’ [for a Code of Practice], because again, it’s incredibly helpful for a regulator to have a defined standard […] Well, we’re capable of doing our work without it; it’s just harder.*

*I don’t know if it would be ‘nice to have’. It would almost have to be—there’s a possibility it could even be in the legislation. Not the approval part, but the requirement to follow.*

*It’s not easy to do, but that’s what I would imagine would be happening in health and other fields, is that standards, templates and ultimately audits are checking up to make sure things are being done the way they’re supposed to be done.*

*Where having some standards and guidelines in place […] allows people to know that there’s a metric against which that process is being measured or being applied. And […] you’re reviewing against a standard. I think that’s always helpful, and I think that should be in place.*

There was disagreement about who should be responsible for developing and/or approving Codes of Practice, standards, or even simple guidelines. Several regulators saw the authors of such documents clearly being public bodies at the federal or joint provincial level. Most regulators, however, proposed that Codes of Practice should be implemented by third parties so that the privacy regulators would still be able to investigate any subsequent breaches or complaints (ie, they would not simultaneously support or certify the implementation of good practices, and also investigate them as that would be a conflict of interest). Similarly, standards were proposed to be developed through national or international standards bodies, or through stakeholder consortia. The certification bodies would be separate from the standards bodies, and they may provide these audits as a commercial offering. This is the set-up, for example, for multiple ISO standards.

In general, regulators in smaller provinces expressed that they do not have the resources to develop guidance documents and, therefore, have to rely on material produced by the larger jurisdictions, or other national or international guidance documents and standards. Currently,^32^ the Ontario IPC has a guidance document on de-identification,^33^ there are recent regulations on the anonymization of personal information from the Government of Quebec,^34^ and an ISO standard on de-identification has been published.^35^ However, there is no national guidance in place in Canada.

### Regulating the anonymized data

#### General views

In almost all instances, the response to the question of whether anonymized data should still be regulated was contingent on the anonymization being done properly. Therefore, that would be the starting assumption in the following summary. Another assumption for this section was that the anonymized data would be used or disclosed for a secondary purpose. There were no specific distinctions made in the responses between different conditions for uses versus disclosures, or whether such disclosures were to commercial entities, open data, public sector organizations, or to academic institutions, for example. Although there are some caveats that are particularly relevant for open data, these are addressed in the ‘Discussion’ section.

Some regulators agreed that there should be a way to control anonymized data. They justified the need for regulation in terms of the remaining ethical considerations. Also, properly anonymized data do not achieve zero identity disclosure risks, even if they are very small, and therefore, they noted that some regulation of anonymized data would be necessary.

*But there should still be some controls in place, I think; some regulation, so that it’s not, basically, a free for all.*

*[Risks of misuse] are ethical issues, in my view, but they’re real.*

Although this view was not unanimous and a minority expressed a different view, few interviewees deemed anonymized data to fall outside privacy legislation. Therefore, there should not be any additional obligations or constraints on the processing of anonymized data, as it is not considered personally identifiable information anymore.

*To me, the only question that really matters is whether there is a serious possibility of re-identification, and if the answer is no, then my view is that regulators then should step back.*

There was a general consensus that certain obligations, such as a prohibition on attempts to re-identify or attack anonymized data, are desirable. This would be considered as a form of regulation of anonymized data.

*[…] if a piece of legislation says, ‘no one can use it for criminal purposes, and no one can use it for fraudulent purposes’, how would you argue against that type of constraint.*

Most interviewees supported the idea of proportionate obligations: The obligations of organizations processing anonymized data should be fewer than the obligations of organizations processing personally identifiable information. Also, some obligations on anonymized data would be very difficult to operationalise in practice, such as deletion and access because the anonymized records cannot be linked back to a specific individual.

#### Restriction on purpose

In our interviews, we introduced the idea of restricted purposes for anonymized data. For example, under Bill C-27, pseudonymised information can be disclosed to certain organizations without an individual’s knowledge or consent if it is for socially beneficial purposes.^36^ Some respondents supported the idea of restricted purposes as a mechanism to oversee anonymized data. According to the interviewees, restriction on purpose should be reasonable and use broad definitions, such as ‘socially beneficial’. At the same time, however, they expressed their concerns that broad terms introduce ambiguity and uncertainty resulting in disincentives for anonymization. In the end, the regulator would determine whether that particular purpose was met on a case-by-case basis.

*So I totally recognize that concepts that are defined at a high level of generality will present operational practical difficulties, but if the alternative is either consent […] or treating all de-identified information the same way regardless of the purpose [then that would not be desirable either] . . . .*

*And if that data is used for a different purpose, to make decisions about people, there [are] fairness considerations, […] there should be some purpose limitations around it, because there might be some unintended consequences.*

*We could say, ‘provided it’s for any socially beneficial purpose’, but then that doesn’t get us very far. Some decision maker […] would still have to decide […].*

#### Transparency and notice

Additional practices were discussed, such as providing notice to data subjects that their information would be anonymized and about how their anonymized data would be used. The general view was that transparency builds trust and establishes a common understanding on how data are used. Regulators rejected the idea of complex transparency mechanisms that required active endorsement by data subjects but supported notice, for example, on the respective website, that is meaningful, easy to read, and to understand.

*I have no sympathy for the point of view that it’s better not to tell people so as not to create any noise. I do not believe that that’s an acceptable public policy stance.*

*There’s nothing to be gained, in my view, by obscuring the fact that there is a point of origin that involved real people.*

*I recognize the difficulty of being transparent more than superficially. The information provided should be, on the level of principle, reader-friendly to the extent possible.*

*Trust is one of the key concepts we’re talking about here […] and I think that public consultation is one way of also helping to achieve trust with the stakeholders at issue, and the actual data subjects.*

*[…] they have that right to know […] I think, [it] is helpful, and there should always be that transparency [with] citizens.*

*Transparency absolutely should happen, but it needs to be meaningful transparency.*

*[A lack of transparency] is that you really impact your own trust level. And subtractions of trust are greater than additions to the trust bank account.*

#### Ethics reviews

The Canadian Tri-Council Policy Statement: Ethical Conduct for Research Involving Human (TCPS) is the official Canadian human research ethics policy of the three federal research funding agencies.^37^ It is a condition of funding to comply with this policy, and research ethics board (REB) review is mandatory for research involving human participants, including research involving anonymized data. ^38^

Organizations that do not receive funding from the three federal research funding agencies, including most of the private sector, may voluntarily choose to follow the TCPS or follow a different ethical framework such as the Consensus Guideline for Good Clinical Practice by the International Council for Harmonization^39^ or the Standard for Ethical Review and Oversight of Human Research by the Human Research Standards Organization.^40^ There are also professional codes that require an REB consultation prior to any research with potential disciplinary actions in case of non-compliance.^41^ Regulatory bodies for medicine and healthcare products, such as Health Canada, the Food and Drug Administration in the USA, and the European Medicines Agency, mandate REB approval for clinical trials,^42^ although these would not be for the processing of anonymized data. Even without any applicable framework, organisations may establish a form of self-governance such as Meta’s Oversight Board for content moderation that, notwithstanding any implementation criticism, exemplifies a voluntary initiative to address ethical concerns.^43^

Therefore, Canada, like many other countries, has various mandatory ethical frameworks complemented by voluntary initiatives, but lacks a uniform requirement to ensure consistent ethical oversight of data processing activities, and specifically for anonymized data used for non-research purposes, in all circumstances. Therefore, privacy regulations may be the primary mechanism for ensuring individual rights.

All regulators in our interviews recognized that there is potentially a moral gap for projects beyond the scope of the TCPS. While privacy regulations may address privacy concerns and data protection, they do not fully consider broader ethical aspects. The processing of anonymized data can cause group harm to individuals even if they are not identifiable. In this sense, performing ethics reviews was positively perceived to ensure that the purposes for processing the anonymized data are not discriminatory, ‘creepy’, surprising, or potentially harmful to the data subjects. There was also the idea that this could be where there is some oversight over anonymized data, but only a few demanded that ethics reviews for non-research projects should be part of the set of obligations and also the issue of ethics-washing was raised.

*I think we need to be looking at it from a population perspective as well. I don’t think you can ignore that fact. So, I definitely think that it’s important to be having ethical reviews of the use of this data, particularly where it’s in the public domain.*

*To have a review based on ethics is a good thing. It should be encouraged; it might be recommended, but I’m a bit concerned with ethics-washing.*

*People are doing research and they’re being regulated, and then people who are possibly using it for marketing or commercial purposes aren’t? That doesn’t seem right.*

*If there is some ethics review, or oversight,[…] it also enables review, which is incredibly important, because that review […] just promotes public confidence.*

*I would suggest that an ethics board review would be one way for an organization to support the fact that their processing is fair and ethical […] but I don’t think we would necessarily be recommending that every use of de-identified data has to go through an ethics review process.*

### Regulation of AIML models

AIML models are known to be susceptible to adversarial attacks that can reveal sensitive information about the individuals in the training datasets.^44^ It means that sharing AIML models that were trained on personally identifiable information could lead to different types of disclosure risks, making (unprotected) AIML models equivalent to the personally identifiable information,^45^ although that view has been challenged in the context of the GDPR.^46^

Interviewees indicated that if a model has identifiable information or some training records that can realistically be recovered and the model (partially) converted to personal information, then it should be treated as such. However, if a machine learning model was trained on anonymized information and even if an adversarial attack is successful, the disclosure risk is low, then the same considerations as for anonymized data would apply to the models.

*If you can attack that in a reasonable way and extract data from it, then it’s a pretty solid argument that that contains personal information.*

Regulators highlighted further risks of models (ie, group harm) but were unsure of how and where to address them adequately.

*So, is the model the right target? Or is it decisions—discriminatory, harmful decisions being made by companies, governments, […]*

*I don’t see how […] we would be able to regulate, to that extent, the technology.*

## Discussion

### Summary

While there was consensus on key points, the regulator’s perspectives were not unanimous, and this resulted in variations in responses to the issues we were investigating. Some general conclusions can be drawn from the interviews as follows.

#### There is not a single Canadian perspective on regulating anonymized data (lack of consistency)

There are different perspectives on regulating anonymized data (including not regulating anonymized data) with different expectations on key aspects across the country. This means that there will be some jurisdictions where it will be easier to apply anonymization and others where this will be more challenging. This is not completely surprising given the heterogeneity in the definitions of personal information and non-identifiable information across the country and across sectors, and how broad these definitions are, as summarized in Supplementary Tables S1, S2, and S3. Furthermore, it remains to be seen whether the recently enacted Quebec regulation respecting the anonymization of personal information^47^ and planned updates to Ontario’s guidance^48^ will affect Canadian privacy regulators’ approach to this subject.

The line between identifiable and non-identifiable is similarly difficult to draw. As shown in Supplementary Tables S1, S2, and S3, the categorisation, if defined, is binary. In practice, however, identifiability can be seen as a spectrum. Several risk thresholds have been proposed for determining an acceptable low risk of identifiability that could match the legal terms.^49^

Binary definitions of identifiability are not limited to the Canadian regulatory framework. For instance, Beduschi describes this in the context of the GDPR.^50^ However, the implications of privacy regulations not being applicable for data that is non-identifiable are non-trivial, as that would allow for an oversight-free processing of these data. At the same time, proceeding on the basis that the residual disclosure risk in anonymized data is not low enough can frustrate other important efforts by the public sector, for example, open data initiatives,^51^ and can create disincentives for anonymization.

For national organizations that end up being regulated differently in multiple jurisdictions, the lack of consistency can create a practical challenge.

#### Fundamental issues are left to interpretation (lack of certainty)

In many jurisdictions across Canada, key elements of anonymization and its implementation are not explicitly addressed in privacy legislation or regulations, resulting in a high level of uncertainty about which perspective to take and much depends on how they are interpreted. Even for issues where there was consensus, such as whether the act of anonymization requires consent, this was based on interpretations rather than being explicit in legislation or regulation (with very few exceptions such as Ontario’s PHIPA). The strong dependence on the interpretation by the incumbent regulator increases the uncertainty with longer-term planning and for long-term investments in data-driven projects requiring anonymization. While there is Canadian guidance in Ontario^52^ and Quebec,^53^ the Ontario guidance (which was published in 2016) was not mentioned during the interviews, except during the Ontario interview, and the Quebec anonymization regulations were finalised after our study was completed and therefore its impact is unknown at this point.

#### Proper anonymization needs to be ensured

Although not all of the interviewees talked explicitly about Codes of Practices, they demanded more verifiable and enforceable guidelines that enable a proactive role including audits or consequences for non-compliance. These requirements would be met by recognised standards and Codes of Practice.

#### Anonymized data should not be left free of oversight

Regulators agreed that there should be a prohibition on attempts to re-identify or attack anonymized data. There was no dominant opinion on what further potential controls should look like. While some regulators preferred the restriction that anonymized data should be processed for socially beneficial purposes, others rejected this idea due to the uncertainty introduced by such terms. Uncertainty potentially results in disincentives for investing in anonymization and have negative consequences for organizations wishing to re-use data for socially beneficial purposes and for legitimate commercial purposes. Softer measures such as recommended transparency notices or ethics reviews were generally favoured but were largely not seen as mandatory.

### Recommendations

Based on the results of the interviews and informed by the principles of reducing uncertainty and creating incentives for the adoption of anonymization, we can make the following recommendations.

#### Develop Code(s) of Practice for proper anonymization

In the interviews, the term Code of Practice was used to refer to an enforceable set of guidelines or rules recognized by privacy regulators, providing concrete guidance to organisations on proper anonymization. Such guidance would make it easier for these organisations to implement anonymization, provide some assurance to regulators that anonymized data were generated properly, and would match the expectations and preferences expressed by the regulators. Important topics to include in such a code would be the definition of precise metrics for evaluating privacy risks and benchmarks for interpreting them, as well as guidance on ensuring the ethical applications of anonymized data, such as setting up an oversight body within an organization for data they anonymize. An ability to demonstrate that such practices have been followed, through the requirement to report specific metrics and possibly through certifications or audits of practices, would be an important requirement in the code.

There is currently no national Canadian privacy legislation in effect that speaks to the development, implementation, approval, and/or oversight by the relevant privacy regulator of Codes of Practice related to the anonymization of personal information. This would change if the provisions of Bill C-27 related to what the bill terms ‘Codes of Practice and Certification Programs’ were to be enacted. However, it merely states that the Commissioner may approve a Code of Practice if they determine that the code meets the criteria set out in the regulations.^54^ Accordingly, it remains for the regulators to determine the definition of ‘Code of Practice’.

Rather than develop a Code of Practice from scratch, which would be a significant undertaking, there are a number of international sources that may be adapted to the Canadian privacy context. For example, the US federal health information privacy legislation, the Health Insurance Portability and Accountability Act (HIPAA) Privacy Rule provides for two methods of de-identification of protected health information: Expert Determination and Safe Harbor. The US Department of Health and Human Services has provided detailed guidance for organizations subject to HIPAA on the methods and approaches to achieve de-identification in accordance with the HIPAA Privacy Rule.^55^ Another more recent guidance document on anonymization was published by the Singapore Personal Data Protection Commission, providing an accessible and applied introduction to the topic.^56^ There are also international standards organizations, such as ISO, that have produced standards applicable to the de-identification of personal information: the new ISO standard, *ISO/IEC 27559:2022 Privacy-enhancing data de-identification framework*,^57^ in combination with the definitional standard—*ISO/IEC 20889:2018 Privacy-enhancing data de-identification terminology and classification of techniques*,^58^ provides both a management and technical framework for the anonymization of personal information.

A remaining question is who should approve such a Code of Practice. There isn’t universal comfort with self-regulation among Canadian regulators; therefore, some form of public sector approval for such a Code of Practice is the likely setup that would be deemed acceptable. It may be a pragmatic approach to have Codes of Practice be sector-specific rather than universally applicable, which would enable the expeditious leveraging and customisation of existing guidelines and standards, as well as acceptance by affected industries and sectors. Multiple codes of practice could be built upon existing sector-specific structures such as industry standards or certification processes. On the other hand, a universal code ensures consistency. A hybrid approach with a universal high-level code and tailored sector-specific ones could combine the strengths of both approaches allowing for consistency and flexibility. Any universal code needs to be developed and acknowledged through a collaborative process involving cross-sectoral and cross-provincial consultation. This may make it more difficult to develop but at the same time more robust and accepted in the long run.

Note that PIAs are another tool to assist Canadian privacy regulators. These are commonly used and give assistance in determining whether entities subject to their oversight have used accepted and verifiable mechanisms to anonymize personal information. There are, however, few Canadian privacy laws that require an entity to conduct a PIA, let alone submit one to the privacy regulator for review and/or approval.^59^ We did not address PIAs in our interviews.

#### Regulatory obligations should be commensurate with the level of risk

If the previous recommendation is followed and anonymization conforms to an established Code of Practice that ensures best practices are followed, then the resulting data will have a very small identity disclosure risk. Under that assumption, it would be logical for the obligations in existing statutes that apply to identifiable data to be reduced for properly anonymized data. This creates an incentive to continuously improve anonymization methods and their application. If the obligations are the same irrespective of how good anonymization is at protecting identifiability, then there would be little incentive to make the investments to develop and adopt better methods.

#### Increase certainty in how anonymization and anonymized data are regulated

While existing statutes across the country are not all precise on some key aspects of regulating anonymization practices and anonymized information, most regulators took a pragmatic approach that balances privacy risks with the benefits of using data in a responsible manner. However, because this state of affairs is dependent on the incumbent regulators, the interpretations of statutes and how they are enforced may change over time. Having precision within the statutes themselves provides certainty to organizations about what the rules to follow are, and in practice, this would be helpful for responsible uses of data. More specifically, this means being explicit on: (i) whether the act of anonymization requires additional consent, (ii) whether anonymity should be assessed from the perspective of the data custodian, the data recipient, or other party,^60^ and (iii) transparency requirements.

Since the risk of identity disclosure cannot be zero (and consequent concerns about re-identification attacks) and there is always some residual identity disclosure risk with anonymized data, some regulators were more comfortable imposing additional obligations, such as purpose limitation or additional consent requirements. Depending on the nature of these constraints, they may create significant disincentives. For example, limiting the use and disclosure of anonymized data to socially beneficial or commercially legitimate purposes would be reasonable because these are broadly defined. However, the broad definitions introduce uncertainty and this would also limit open data initiatives because a data custodian cannot guarantee that these purposes will be met.

#### Ethical considerations should be addressed

While properly anonymized data have very low identity disclosure risk, there is still potential harm that might be more indirect and not related to the privacy of the individual, but rather to the individual belonging to a group.^61^ This can be labelled as group or collective privacy.^62^ In this context, harm can result from predictive modelling where conclusions are drawn about groups of people. Consequences may be aggravated biases, health disparities, systematic inequality, and misuse.^63^ A research project by British Columbia’s Office of the Human Rights Commissioner pointed out that while information about groups may foster equality and justice, it can also ‘reinforce the idea that individuals and groups are to blame for their own marginalization by portraying them as lacking in some way or implying that identities are grounded in innate biological differences rather than making them visible as social constructions’.^64^ They list examples of information about groups that could be used to address systematic inequalities while carrying the risk of stigmatization such as ‘Some 56 per cent of Black Canadians report layoffs or reduced working hours during COVID-19’, ‘Only 25 per cent of Indigenous communities in B.C. have basic internet access” or “More than 40 per cent of homeless youth are LGBTQ2S+’.^65^ This information could have potentially been drawn from anonymized data. In this sense, all regulators recognized that ethical considerations are orthogonal to identifiability. Potentially harmful consequences beyond individual privacy disclosure are part of ethical reviews, but there is a moral gap for projects that do not explicitly fall within the scope of traditional REB reviews (see Ethics reviews Section).

This moral gap should be addressed to ensure fair and ethical processing of information, which could be achieved through a required and proportionate ethics oversight or an ethical impact assessment. Having a form of ethics review can substantially help to develop mitigation strategies and, ultimately, prevent individual and group harm.

### Limitations

Having achieved a high coverage among privacy regulators in Canada, we created a comprehensive picture of their perspectives and, based on that, formulated recommendations that could enable the broader use of responsible anonymization practices and the use and disclosure of anonymized data in Canada. Nevertheless, there are limitations in the study and our reporting of it to note: (i) we agreed to interchangeably use the terms ‘anonymized’ and ‘de-identified’ to refer to information for which there is no serious possibility in the circumstances that it could be associated with an identifiable individual; however, these terms are used to connote different things across sectors and provinces which may have led to divergent understandings of the interview questions, (ii) our recommendations are specific to current Canadian legislation and are based on interviews with Canadian regulators at the time the interviews were conducted and therefore may not be generalisable to other regulatory frameworks, (iii) even if we have covered almost the whole potential interview population, it is possible that we have missed certain aspects due to the one missing jurisdiction, and (iv) our interpretation of qualitative data was informed by grounded theory but remains subjective and might have been influenced by the researchers’ preconceptions and experiences.

## Conclusions

The definitions of anonymized information and the process of anonymization are inconsistent and often unclear on key parameters among privacy legislation, regulations, and guidelines across Canada. We conducted interviews with 93 per cent of Canadian privacy regulators to understand their views on how anonymized data and anonymization currently are, and should be, regulated. We identified some important areas of consensus, such as there being no requirement to obtain consent for the act of anonymization and the need for Code(s) of Practice; areas of divergence, such as imposing purpose constraints on processing anonymized data; and areas of uncertainty, such as how to regulate AIML models trained on personally identifiable information versus anonymized information. Recommendations were formulated to operationalize the areas of consensus and to clarify areas of divergence and uncertainty.

## Ethics

This study was approved by the CHEO Research Institute REB protocol CHEOREB# 22/08X. The research was performed in accordance with relevant guidelines/regulations for human subjects research in Canada.

## Supplementary Material

ipae017_Supplementary_Data

## Footnotes

1 Tjeerd van der Ploeg, Peter C Austin, and Ewout W Steyerberg, ‘Modern Modelling Techniques Are Data Hungry: A Simulation Study for Predicting Dichotomous Endpoints’ (2014) 14 BMC Medical Research Methodology 137.

2 Government Accountability Office, *Artificial Intelligence: Emerging Opportunities, Challenges, and Implications* (March 2018).

3 McKinsey Global Institute, *Artificial Intelligence: The Next Digital Frontier ?* (June 2017).

4 Deloitte Insights, *State of AI in the Enterprise* (2nd edn, 2018).

5 Ben Lorica and Paco Nathan, *The State of Machine Learning Adoption in the Enterprise* (O’Reilly 2018).

6 Willem G van Panhuis and others, ‘A Systematic Review of Barriers to Data Sharing in Public Health’ (2014) 14 BMC Public Health 1144; Shona Kalkman and others, ‘Responsible Data Sharing in International Health Research: A Systematic Review of Principles and Norms’ (2019) 20 BMC Medical Ethics 21.

7 Expert Advisory Group, *Pan-Canadian Health Data Strategy: Building Canada’s Health Data Foundation: Report 2* (Public Health Agency of Canada 2021).

8 <https://www.parl.ca/legisinfo/en/bill/44-1/c-27> Accessed 20 March 2022.

9
*PHIPA Decision 175*, Health Information and Privacy (Office of the Information and Privacy Commissioner of Ontario 25 March 2022) <https://decisions.ipc.on.ca/ipc-cipvp/phipa/en/item/520967/index.do> Accessed 1 April 2022.

10 Office of the Privacy Commissioner of Canada, *Investigation into the Collection and Use of De-Identified Mobility Data in the Course of the COVID-19 Pandemic* (Office of the Privacy Commissioner of Canada 30 May 2023) <https://www.priv.gc.ca/en/opc-actions-and-decisions/investigations/investigations-into-federal-institutions/2022-23/pa_20230529_phac/> Accessed 1 June 2023.

11 Office of the Information and Privacy Commissioner of Ontario, *Deidentification Guidelines for Structured Data* (Office of the Information and Privacy Commissioner of Ontario 2016) <http://bit.ly/2ypH3B1> Accessed 1 March 2022.

12 Gazette Officielle du Quebec, 156, 1758–1760, 15 May 2024. See <https://bit.ly/3VS5K26> Accesed 30 May 2024.

13 Future de-identification regulations under PHIPA may, however, align it with the Quebec law.

14 Office of the Privacy Commissioner of Canada, *Submission of the Office of the Privacy Commissioner of Canada on Bill C-27, the Digital Charter Implementation Act, 2022*, 2023 <https://www.priv.gc.ca/en/opc-actions-and-decisions/submissions-to-consultations/sub_indu_c27_2304/> Accessed 1 May 2023.

15 Gazette Officielle du Quebec (n 12).

16 Article 29 Data Protection Working Party, *Opinion 05/2014 on Anonymization Techniques*, 10 April 2014.

17 Kelly E Ormond and others, ‘What Are the Bottlenecks to Health Data Sharing in Switzerland? An Interview Study’ (2024) 154 *Swiss Medical Weekly* 3538; Raphaël Chevrier and others, ‘Use and Understanding of Anonymization and De-Identification in the Biomedical Literature: Scoping Review’ (2019) 21 Journal of Medical Internet Research e13484.

18 Canadian Anonymization Network (CANON), *States of Data* 2020 <https://deidentify.ca/wp-content/uploads/2020/10/CANON-States-of-Data-One-Pager.pdf> Accessed 1 March 2022.

19 To map these terms to terms that have been introduced in recent legislation, Bill C-27 uses the term ‘de-identification’ to mean ‘pseudonymization’ in our definition, and the term ‘anonymization’ to mean ‘anonymization’ or ‘de-identification’ in our definitions. This is similar to the definitions in the Quebec *Act Respecting the Protection of Personal Information in the Private Sector*.

20 Scott P. Layne, James M. Hyman, David M. Morens and Jeffery K. Taubenberger, ‘New Coronavirus Outbreak: Framing Questions for Pandemic Prevention’ (2020) 12, eabb1469 Science Translational Medicine; Moira Downey, *Sharing Data and Research in a Time of Global Pandemic* (Duke University Libraries 2020) <https://blogs.library.duke.edu/bitstreams/2020/03/17/sharing-data-and-research-in-a-time-of-global-pandemic/> accessed 8 April 2020; Alfred Ng, ‘Coronavirus Pandemic Changes How Your Privacy Is Protected’ *CNET* 2020 <https://www.cnet.com/news/coronavirus-pandemic-changes-how-your-privacy-is-protected/> Accessed 22 March 2020.

21 George Gaskell, ‘Individual and Group Interviewing’ in *Qualitative Researching with Text, Image and Sound: A Practical Handbook* (SAGE Publications 2000) 38–56 <https://methods.sagepub.com/book/qualitative-researching-with-tet-image-and-sound/n3.xml>; John W. Creswell and Cheryl N. Poth, *Qualitative Inquiry and Research Design: Choosing Among Five Approaches* (4th ed, SAGE Publications 2018) <https://us.sagepub.com/en-us/nam/qualitative-inquiry-and-research-design/book246896>

22 Creswell and Poth, ibid.

23 Gaskell (n 21); Creswell and Poth (n 21).

24 Gaskell (n 21).

25 ibid

26 ibid.

27 J. Creswell, *Research Design: Qualitative, Quantitative, and Mixed Methods Approaches* (Sage 2003); J Creswell and V Plano-Clark, *Designing and Conducting Mixed Methods Research* (Sage 2007).

28 Creswell and Plano-Clark, ibid.

29 See <https://www.priv.gc.ca/en/opc-actions-and-decisions/investigations/investigations-into-businesses/2018/pipeda-2018-003/> Accessed 1 March 2022.

30 See ‘Synthetic Data White paper’, <https://healthcities.ca/synthetic-data-white-paper/> Accessed 2 July 2023.

31 See Ontario’s PHIPA s 37(1)(f).

32 Some of these documents were published after the completion of the interviews.

33 Information and Privacy Commissioner of Ontario, *De-Identification Guidelines for Structured Data* (June 2016) <http://bit.ly/1PkrnMF> Accessed 1 March 2022.

34 Gazette Officielle du Quebec (n 12).

35
*ISO/IEC 27559:2022: Information Security, Cybersecurity and Privacy Protection—Privacy Enhancing Data de-Identification Framework*, November 2022 <https://www.iso.org/standard/71677.html> Accessed 5 January 2023.

36 See s 39(1) of Bill C-27; also under the Quebec Act Respecting the Protection of Personal Information in the Private Sector, anonymization is an alternative to data destruction, but only if the data are used for ‘serious and legitimate purposes’ (see s 23); however, this specific purpose constraint did not come up during our interviews.

37 Panel on Research Ethics, *Tri-Council Policy Statement: Ethical Conduct for Research Involving Humans* (2022) <https://ethics.gc.ca/eng/policy-politique_tcps2-eptc2_2022.html> Accessed 31 March 2023.

38 There are exceptions, eg, for exploratory phases of research studies, quality improvement studies, programme evaluation activities, performance reviews, testing within normal educational requirements when used exclusively for assessment, and some studies that use public information.

39 U.S. Department of Health and Human Services and Food and Drug Administration, ‘ICH Harmonized Guideline Integrated Addendum to ICH E6(R1): Guideline for Good Clinical Practice ICH E6(R2) ICH Consensus Guideline’ (HHS and FDA, 2016) <https://database.ich.org/sites/default/files/E6_R2_Addendum.pdf> Accessed 5 January 2022.

40 ‘Published Standards—HRSO’, 2020 <https://www.hrso-onrh.org/standards/published-standards/> accessed 24 June 2024.

41 Eg, The ‘CMA Code of Ethics and Professionalism’ (Canadian Medical Association) <https://www.cma.ca/cma-code-ethics-and-professionalism> Accessed 13 November 2023.

42 ‘21 CFR 56.103—Circumstances in Which IRB Review Is Required’, p 21 <https://www.ecfr.gov/current/title-21/part-56/section-56.103> Accessed 1 March 2022; Health Canada, *Research Ethics Board: Policies, Guidelines and Resources* (2021) <https://www.canada.ca/en/health-canada/services/science-research/science-advice-decision-making/research-ethics-board/policy-guidelines-resources.html>Accessed 1 March 2022; *Regulation (EU) No 536/2014 of the European Parliament and of the Council of 16 April 2014 on Clinical Trials on Medicinal Products for Human Use, and Repealing Directive 2001/20/EC Text with EEA Relevance*, *OJ L*, 2014, clviii <http://data.europa.eu/eli/reg/2014/536/oj/eng> Accessed 1 March 2022.

43 David Wong and Luciano Floridi, ‘Meta’s Oversight Board: A Review and Critical Assessment’ (2023) 33 Minds and Machines 261–84.

44 Emiliano De Cristofaro, ‘A Critical Overview of Privacy in Machine Learning’ (2021) 19 IEEE Security & Privacy 19–27; Ahmed Shafee and Tasneem A Awaad, ‘Privacy Attacks against Deep Learning Models and Their Countermeasures’ (2021) 114 Journal of Systems Architecture 101940.

45 Michael Veale, Reuben Binns, and Lilian Edwards, ‘Algorithms That Remember: Model Inversion Attacks and Data Protection Law’ (2018) 376 Philosophical Transactions of the Royal Society A: Mathematical, Physical and Engineering Sciences 20180083.

46 M R Leiser and Francien Dechesne, ‘Governing Machine-Learning Models: Challenging the Personal Data Presumption’ (2020) 10 International Data Privacy Law 187–200.

47 Gazette Officielle du Quebec (n 12).

48 ‘See Embarking on My New Journey as the IPC’s Scholar-in-Residence | Information and Privacy Commissioner of Ontario’ <https://www.ipc.on.ca/en/media-centre/blog/embarking-my-new-journey-ipcs-scholar-residence> Accessed 2 May 2024.

49 Health Canada, *Guidance Document on Public Release of Clinical Information*, 2019 <https://www.canada.ca/en/health-canada/services/drug-health-product-review-approval/profile-public-release-clinical-information-guidance.html>Accessed 1 March 2022; *ISO/IEC 27559* (n 35). <https://www.iso.org/standard/71677.html>

50 Ana Beduschi, ‘Synthetic Data Protection: Towards a Paradigm Change in Data Regulation?’ (2024) 11 Big Data & Society 20539517241231277.

51 ibid.

52 Office of the Information and Privacy Commissioner of Ontario (n 11).

53 Gazette Officielle du Quebec (n 12).

54 Bill C-27, s 76(3).

55 ‘Guidance Regarding Methods for De-identification of Protected Health Information in Accordance with the Health Insurance Portability and Accountability Act (HIPAA) Privacy Rule’ <https://www.hhs.gov/hipaa/for-professionals/privacy/special-topics/de-identification/index.html> Accessed 1 March 2022.

56 PDPC, *Guide to Basic Anonymization* (2022) <https://www.pdpc.gov.sg/news-and-events/announcements/2022/03/guide-to-basic-anonymization-now-available> Accessed 1 April 2022.

57 ISO/IEC 27559: 2022 (n 35).

58 ISO/IEC 20889:2018—Privacy enhancing data de-identification terminology and classification of techniques.

59 Eg, while s 17 of the new Quebec private sector law requires an organization to conduct a PIA prior to communicating personal information outside of the province, these do not require that the PIA be submitted to the privacy regulator. s 64 of the Alberta *Health Information Act* requires custodians to prepare and submit to the Commissioner’s office a PIA ‘that describes how proposed administrative practices and information systems relating to the collection, use and disclosure of individually identifying health information may affect the privacy of the individual who is the subject of the information.’ However, the Commissioner’s role is limited to ‘review and comment’, and there is no obligation on the custodian to make any changes related to its practices based on the feedback provided by the Commissioner. Bill C-27 does not contain any provisions requiring an organization to conduct a PIA.

60 Lore Leitner, Gabe Maldoff, and Mickey Lee, ‘Anonymization through Separation: What Recent Cases Can Teach Us about the EU’s Anonymization Standards’ (2024) 24 Privacy and Data Protection 10–13.

61 Agata Ferretti and others, ‘Big Data, Biomedical Research, and Ethics Review: New Challenges for IRBs’ (2020) 42 Ethics & Human Research 17; Simson L. Garfinkel, ‘Beyond IRBs: Designing Ethical Review Processes for Big Data Research’ (presented at the Beyond IRBs, Future of Privacy Forum 2015) <https://bigdata.fpf.org/papers/beyond-irbs-designing-ethical-review-processes-for-big-data-research/>Accessed 1 March 2022.

62 Alessandro Mantelero, ‘From Group Privacy to Collective Privacy: Towards a New Dimension of Privacy and Data Protection in the Big Data Era’ in Linnet Taylor, Luciano Floridi, and Bart van der Sloot (eds), *Group Privacy: New Challenges of Data Technologies* (Springer International Publishing 2017) 139–58; Rainer Mühlhoff, ‘Predictive Privacy: Towards an Applied Ethics of Data Analytics’ (2021) 23 Ethics and Information Technology 675–90; Mantelero, ibid.

63 Ziad Obermeyer and others, ‘Dissecting Racial Bias in an Algorithm Used to Manage the Health of Populations’ (2019) 366 Science 447–53.

64 British Columbia’s Office of the Human Rights Commissioner, *Disaggregated Data: Summary of Recommendations to Prevent Harm to Communities* (2021).

65
*Disaggregated Data Collection in B.C.: The Grandmother Perspective* (BC’s Office of the Human Rights Commissioner 2020) <https://bchumanrights.ca/publications/datacollection/> Accessed 1 March 2022.

