# Supplementary material for: Perspectives of Canadian privacy regulators on anonymization practices and anonymized information: a qualitative study
Source: Int Data Priv Law. 2024 Dec 18;14(4):391–403. doi: 10.1093/idpl/ipae017 (PMC11970032; doi:10.1093/idpl/ipae017)
Supplement: ipae017_Supplementary_Data [file ipae017_supplementary_data.pdf]

Appendix A: Definition of Personal Information / Non-identifiable information

The following tables summarize the definitions used in Canadian legislation for personal information.

PRIVATE SECTOR				
**Legislation**	**Jurisdiction**	**Definition of Personal Information**	**Definition of "Identifiable"?**	**Other Provisions?**
Personal Information Protection and Electronic Documents Act (PIPEDA)	Federal	Information about an **identifiable** individual…	_	_

Bill C-27: The Consumer Privacy Protection Act	Federal	information about an **identifiable i**ndividual	**de-identify** means to modify personal information so that an individual cannot be **directly identified from it, though a risk of the individual being identified remains**<br><br>**anonymize** means to irreversibly and permanently modify personal information, in accordance with generally accepted best practices, to ensure that no individual can be identified from the information, whether directly or indirectly, by any means	**Socially beneficial purposes**<br>39 (1) An organization may disclose an individual's personal information without their knowledge or consent if<br><br>(a) the personal information is de-identified before the disclosure is made;<br><br>(b) the disclosure is made to<br><br>(i) a government institution or part of a government institution in Canada,<br><br>(ii) a health care institution, post-secondary educational institution or public library in Canada,<br><br>(iii) any organization that is mandated, under a federal or provincial law or by contract with a government institution or part of a government institution in Canada, to carry out a socially beneficial purpose, or<br><br>(iv) any other prescribed entity; and<br><br>(c) the disclosure is made for a socially beneficial purpose.<br><br>**Definition of socially beneficial purpose**<br>(2) For the purpose of this section, socially beneficial purpose means a purpose related to health, the provision or improvement of public amenities or infrastructure, the protection of the environment or any other prescribed purpose.<br><br>**De-identification of Personal Information**<br>74 An organization that de-identifies personal information must ensure that

				any technical and administrative measures applied to the information are proportionate to the purpose for which the information is de-identified and the sensitivity of the personal information.<br><br>**Prohibition**<br>75 An organization must not use information that has been de-identified, alone or in combination with other information, to identify an individual except<br><br>(a) to conduct testing of the effectiveness of security safeguards that it has put in place;<br><br>(b) to comply with any requirements under this Act or under federal or provincial law;<br><br>(c) to conduct testing of the fairness and accuracy of models, processes and systems that were developed using information that has been de-identified;<br><br>(d) to conduct testing of the effectiveness of its de-identification processes;<br><br>(e) for a purpose or situation authorized by the Commissioner under section 116; and<br><br>(f) in any other prescribed circumstance.
Personal Information Protection Act (Alberta PIPA)	Alberta	Information about an **identifiable** individual	_	_

Personal Information Protection Act (BC PIPA)	British Columbia	Information about an **identifiable** individual and<br>includes employee personal information but does not include<br>(a) contact information, or<br>(b) work product information;		
An Act respecting the protection of personal information in the private sector	Quebec	Any information which relates to a natural person and allows that person to be identified	For the purposes of this Act, personal information is<br>(1) de-identified if it no longer allows the person concerned to be directly identified;<br><br>For the purposes of this Act, information concerning a natural person is anonymized if it irreversibly no longer allows the person to be identified directly or indirectly.<br><br>Information anonymized under this Act must be anonymized according to generally accepted best practices."	**O.C. 783-2024, 1 May 2024**<br>Act respecting access to documents held by public bodies and the protection of personal information<br>(chapter A-2.1)<br>Act respecting the protection of personal information in the private sector<br>(chapter P-39.1)<br>Anonymization of personal information<br>Regulation respecting the anonymization of personal information

Table S1: Definition of identifiable information in general Canadian privacy legislation.

HEALTH INFORMATION SPECIFIC				
**Legislation**	**Jurisdiction**	**Definition of Personal Health Information**	**Definition of "Identifiable"?**	**Other Provisions?**
Health Information Act (HIA)	Alberta		"individually identifying", when used to describe health information, means that the identity of the individual who is the subject of the information can be readily ascertained from the information;	**Collection** of non-identifying health information<br><br>19 A custodian may collect non-identifying health information for any purpose.
			"non-identifying", when used to describe health information, means that the identity of the individual who is the subject of the information cannot be readily ascertained from the information;	**Use** of non-identifying health information<br><br>26 A custodian may use non-identifying health information for any purpose.
				**Disclosure** of non-identifying health information<br><br>32(1) A custodian may disclose non-identifying health information for any purpose
				Power to transform health information<br><br>65 A custodian may, in accordance with the regulations, strip, encode or otherwise transform individually identifying health information to create non-identifying health information.
				**Regulations:**<br><br>108(1) The Lieutenant Governor in Council may make regulations<br>(i) respecting the stripping, encoding or other transformation of individually identifying health information to create non-identifying health information pursuant to section 65 or an agreement referred to in section 66; **Note that there are currently no regulations made pursuant to this section.**

HEALTH INFORMATION SPECIFIC				
**Legislation**	**Jurisdiction**	**Definition of Personal Health Information**	**Definition of "Identifiable"?**	**Other Provisions?**
Personal Health Information Act (PHIA)	Manitoba	recorded information about an identifiable individual that relates to…and includes …e) any identifying information about the individual that is collected in the course of, and is incidental to, the provision of health care or payment for health care;	–	**Application of this Act**<br><br>3 This Act does not apply to anonymous or statistical health information that does not, either by itself or when combined with other information available to the holder, permit individuals to be identified.

HEALTH INFORMATION SPECIFIC				
**Legislation**	**Jurisdiction**	**Definition of Personal Health Information**	**Definition of "Identifiable"?**	**Other Provisions?**
Personal Health Information Privacy and Access Act	New Brunswick	"personal health information" means identifying information about an individual in oral or recorded form …	**"de-identified"** , when referring to personal health information, means personal health information from which all identifying information has been removed<br><br>**identifying information"** means information that identifies an individual or for which it is reasonably foreseeable in the circumstances that it could be utilized, either alone or with other information, to identify an individual	**Application of the Act**<br>Unless otherwise specifically provided in this Act, this Act does not apply to<br>(a) anonymous or statistical information that does not, either by itself or when combined with other information available to the holder of the information, permit individuals to be identified,<br><br>A custodian may collect personal health information that has been de-identified for any purpose.<br><br>A custodian may use personal health information that has been de-identified for any purpose.<br><br>A custodian may use personal health information in its custody or under its control for one or more of the following purposes:<br>for the purpose of disposing of the information or de-identifying the information;<br>to produce de-identified information that does not, either by itself or in combination with other information in the custody of or under the control of the custodian, permit an individual to be identified.<br><br>A custodian may disclose personal health information that has been de-identified for any purpose.<br><br>**Power to transform personal health information**<br>A custodian may strip, encode or otherwise transform personal health information in order to create or produce de-identified information.

HEALTH INFORMATION SPECIFIC				
**Legislation**	**Jurisdiction**	**Definition of Personal Health Information**	**Definition of "Identifiable"?**	**Other Provisions?**
Personal Health Information Act	Newfoundland and Labrador	"personal health information" means identifying information in oral or recorded form about an individual that relates to ….	**"identifying information"** means information that identifies an individual or for which it is reasonably foreseeable in the circumstances that it could be utilized, either alone or together with other information, to identify an individual.	**Power to transform personal health information**<br>A custodian may strip, encode or otherwise transform personal health information to create non-identifying health information.<br><br>A custodian may use personal health information in its custody or under its control for one or more of the following purposes:<br><br>to produce information that does not, either by itself or in combination with other information in the custody of or under the control of the custodian, permit an individual to be identified.
Health Information Act	Northwest Territories	"personal information" means information in any form that identifies an individual, or in respect of which it is reasonably foreseeable in the circumstances that the information could be used, either alone or with other information, to identify an individual		Nothing in this Act shall be construed so as to identifying prevent a health information custodian from collecting, health information using or disclosing non-identifying information<br>A health information custodian may use personal custodian health information about an individual …to produce information that does not permit an individual to be identified;<br>Subject to the regulations, a health information custodian may strip, encode or otherwise information transform personal health information to create or produce non-identifying information.<br>Regulation<br>The administrative, technical and physical safeguards required under section 85 of the Act must include (a) measures to protect personal health information through an assessment of re-identification risk and the application of de-identification procedures as require

HEALTH INFORMATION SPECIFIC				
**Legislation**	**Jurisdiction**	**Definition of Personal Health Information**	**Definition of "Identifiable"?**	**Other Provisions?**
Personal Health Information Act	Nova Scotia	"personal health information" means identifying information about an individual, whether living or deceased, and in both recorded and unrecorded forms, if the information	"identifying information" means information that identifies an individual or, where it is reasonably foreseeable in the circumstances, could be utilized, either alone or with other information, to identify an individual;	"de-identified information" is information that has had all identifiers removed that (i) identify the individual, or (ii) where it is reasonably foreseeable in the circumstances, could be utilized, either alone or with other information, to identify the individual;<br>**Application of the Act**<br>This Act does not apply to (a) statistical, aggregate or de-identified health information; …<br>Subject to Section 50, personal health information may be de-identified and retained for purposes other than the original purposes for which it was collected.
Personal Health Information Protection Act, 2004 (PHIPA)	Ontario	"personal health information", subject to subsections (3) and (4), means identifying information about an individual in oral or recorded form, if the information…	**Identifying information**<br>(2) In this section, "identifying information" means information that identifies an individual or for which it is reasonably foreseeable in the circumstances that it could be utilized, either alone or with other information, to identify an individual	"de-identify", in relation to the personal health information of an individual, means to remove any information that identifies the individual or for which it is reasonably foreseeable in the circumstances that it could be utilized, either alone or with other information, to identify the individual, and "de-identification" has a corresponding meaning;<br>**NOTE:** On a day to be named by proclamation of the Lieutenant Governor, the definition of "de-identify" in section 2 of the Act is amended by striking out "to remove any information" and substituting **"to remove, in accordance with such requirements as may be prescribed, any information"**<br>**A health information custodian may use personal health information about an individual…**<br>(f) in a manner consistent with Part II, for the purpose of disposing of the information or modifying the information in order to conceal the identity of the individual;

HEALTH INFORMATION SPECIFIC				
**Legislation**	**Jurisdiction**	**Definition of Personal Health Information**	**Definition of "Identifiable"?**	**Other Provisions?**
Health Information Act	Prince Edward Island	personal health information" means identifying information about an individual in oral or recorded form that	"identifying information" means information that identifies an individual or which it is reasonably foreseeable in the circumstances could be utilized, either alone or with other information, to identify an individual	**"de-identified information"** means personal health information that has been stripped, encoded or otherwise transformed so as to ensure that the identity of the individual who was the subject of the personal health information cannot be readily ascertained from the de-identified information**;**<br>Unless otherwise specifically provided in this Act, this Act does not apply to (a) anonymous or statistical information that does not, either by itself or when combined with other information available to the holder of the information, permit individuals to be identified<br>A custodian may collect for any purpose personal health information that has been de-identified.<br>A custodian may use for any purpose personal health information that has been de-identified.<br>A custodian may use personal health information in its custody or under its control for one or more of the following purposes:<br>for the purpose of disposing of the personal health information or de-identifying the personal health information;<br>to produce de-identified information<br>A custodian may disclose for any purpose personal health information that has been de-identified.<br>A custodian may strip, encode or otherwise transform personal health information in order to create or produce de-identified information

HEALTH INFORMATION SPECIFIC				
**Legislation**	**Jurisdiction**	**Definition of Personal Health Information**	**Definition of "Identifiable"?**	**Other Provisions?**
The Health Information Protection Act (HIPA)	Saskatchewan		**"de-identified personal health information"** means personal health information from which any information that may reasonably be expected to identify an individual has been removed;	**This Act does not apply to**:<br>(a) statistical information or de-identified personal health information that cannot reasonably be expected, either by itself or when combined with other information available to the person who receives it, to enable the subject individuals to be identified;<br>23(1)(4) A trustee must, where practicable, use or disclose only de-identified personal health information if it will serve the purpose.<br>**Restrictions on use:**<br>**26**(1) A trustee shall not use personal health information in the custody or control of the trustee except with the consent of the subject individual or in accordance with this section.<br>(2) A trustee may use personal health information:<br>(b) for the purposes of de-identifying the personal health information;
Health Information Privacy And Management Act	Yukon	"health information" of an individual means identifying information of the individual, in unrecorded or recorded form, that …	**"identifying information"** of an individual means information that identifies the individual or that it is reasonable to believe could be used, either alone or with other information, to identify the individual;	Nothing in this Act limits any person's right to collect, use or disclose information that is not identifying information.<br>A custodian may, without an individual's consent, use the individual's personal health information that is in its custody or control<br>for the purpose of modifying (including removing identifying information from), destroying or disposing of the information;

Table S2: Definition of identifiable information in health specific Canadian provincial privacy legislation.

PUBLIC SECTOR				
**Legislation**	**Jurisdiction**	**Definition of Personal Information**	**Definition of "Identifiable"?**	**Other Provisions?**
Privacy Act	Federal	information about an identifiable individual that is recorded in any form including, without restricting the generality of the foregoing,…	_	_
Freedom of Information and Protection of Privacy Act (FOIP)	Alberta	…recorded information about an identifiable individual, including…	_	_
Freedom of Information and Protection of Privacy Act	British Columbia	…recorded information about an identifiable individual other than contact information;	_	_
Freedom of Information and Protection of Privacy Act	Manitoba	…recorded information about an identifiable individual, including…	_	_
Right to Information and Protection of Privacy Act	New Brunswick	"personal information" means recorded information about an identifiable individual, …	identifying information" means information that identifies an individual or which it is reasonably foreseeable in the circumstances could be utilized, either alone or with other information, to identify an individual.	A public body may use personal information only_<br>for the purpose of producing de-identified information that does not, either by itself or in combination with other information in the custody or under the control of the public body, permit an individual to be identified.
Access to Information and Protection of Privacy Act, 2015	Newfoundland and Labrador	recorded information about an identifiable individual, including…	_	_
Freedom of Information and Protection of Privacy Act	Nova Scotia	.. recorded information about an identifiable individual, including…	_	_
Part XX of the Municipal Government Act (The Municipal Freedom of Information and Protection of Privacy Act)	Nova Scotia	…recorded information about an identifiable individual, including…	_	_
Freedom of Information and Protection of Privacy Act	Ontario	recorded information about an identifiable individual, including,…	_	_
Municipal Freedom of Information and Protection of Privacy Act	Ontario	recorded information about an identifiable individual, including,…	_	_
Freedom of Information and Protection of Privacy Act	Prince Edward Island	… recorded information about an identifiable individual, including…	_	_

PUBLIC SECTOR				
**Legislation**	**Jurisdiction**	**Definition of Personal Information**	**Definition of "Identifiable"?**	**Other Provisions?**
An Act respecting Access to documents held by public bodies and the Protection of personal information	Quebec	In any document, information concerning a natural person which allows the person to be identified is personal information.	–	–
Freedom of Information and Protection of Privacy Act	Saskatchewan	…about an identifiable individual that is recorded in any form, …	–	–
Local Authority Freedom of Information and Protection of Privacy Act	Saskatchewan	…about an identifiable individual that is recorded in any form, …	–	–

Table S3: Definition of identifiable information in Canadian public sector privacy legislation.

Appendix B: Overview of The Canadian Privacy Landscape

The Canadian privacy law landscape reflects the division of powers between the federal and provincial governments set out in the Constitution. Relevant to this paper, pursuant to the Constitution, the federal government has jurisdiction over matters related to trade and commerce and the provinces have the constitutional authority to regulate matters related to property and civil rights, as well as those related to health care.[1] As a result, the existent Canadian laws may be characterized as those covering the private sector (i.e., the collection, use and disclosure of personal information in the course of commercial activities); the public sector (i.e., government institutions as well as what is called the broader public sector in Canada – such as hospitals that receive their funding from the provincial government); and the health sector which covers entities that operate in both the public and private sector and deliver health services as defined in the relevant legislation.

The Private Sector

The federal privacy legislation the *Personal Information Protection and Electronic Documents Act*[2] (PIPEDA) was enacted by the federal government and came into force in April of 2000. It applies to organizations that collect, use or disclose personal information in the course of commercial activities, federal works and undertakings as well as the transfer of personal information between provinces and outside of Canada. Given that PIPEDA was enacted under the federal government's constitutional trade and commerce powers, PIPEDA is characterized as "private sector" privacy legislation in the context of the Canadian privacy landscape.

In an effort to encourage the provinces to enact their own private sector privacy legislation, PIPEDA initially included a transition period (i.e., a period of time by which a province could enact provincial privacy legislation that was deemed to be "substantially similar"[3] to PIPEDA), in which case the provincial law would apply to entities that collected, used and/or disclosed personal information with that province. To date, the private sector privacy laws of the provinces of British Columbia[4], Alberta[5] and Quebec[6] have been deemed to be "substantially similar" to PIPEDA.

[1] The federal government does have limited jurisdictional authority to regulate health care in limited circumstances such as those related to federally-regulated entities such as inter-provincial transportation as well as First Nations, Inuit and Metis individuals.
[2] S.C. 2000, c.5.
[3] See https://www.priv.gc.ca/en/privacy-topics/privacy-laws-in-canada/the-personal-information-protection-and-electronic-documents-act-pipeda/r_o_p/prov-pipeda/.
[4] The *Personal Information Protection Act,* S.B.C. 2003, c 63 ("B.C. PIPA").
[5] The *Personal Information Protection Act*, S.A. 2003, c P-6.5 ("Alberta PIPA").
[6] An *Act respecting the protection of personal information in the private sector,* CQLR c P-39.1 (the "Quebec Act").

Nonetheless, the regulation of the collection, use and disclosure of personal information by organizations in the course of Canadian commercial activities, is, as is the case in many jurisdictions, in flux, to a large degree as a result of the EU General Data Protection Regulation (the GDPR) and PIPEDA maintaining its "equivalency" status as far as international transfers of personal data from the EU to Canada are concerned. In June of 2022 the federal government introduced the *Digital Charter Implementation Act, 2022* [7](known as Bill C-27) which, among other matters would substantially amend PIPEDA and would rename it as the *Consumer Privacy Protection Act* (the CPPA).

The three provinces that have private sector privacy laws designated as being substantially similar to PIPEDA – British Columbia, Alberta and Quebec - have been so designated according to a set of criteria set out in Footnote 3. The new Quebec private sector law (Bill 64) – which came into effect in three phases, the final one being on September 22, 2024, and has technically not undergone a "substantially similar" review. However, given that the Quebec Act is modeled after the GDPR and this is what the federal government hopes to achieve with Bill C-27, it is expected that the new Quebec private sector law will maintain its substantially similar designation to any PIPEDA amendments that may eventually be enacted in Bill C-27.

The Health Sector

Eight provinces - Alberta, Manitoba, New Brunswick, Newfoundland and Labrador, Nova Scotia, Ontario, Prince Edward Island and Saskatchewan, as well as the Northwest Territories and the Yukon have enacted provincial health information specific privacy laws which apply to "custodians" or "trustees" that process personal health information. These health information sector specific privacy laws recognize that in the Canadian context health services may involve both the public/broader public sector (e.g., government ministries, hospitals) as well as the private sector (e.g., pharmacies, health service providers/ practices) and therefore cover both sectors; the focus is on the entities that process personal health information for certain purposes, rather than the sector in which they operate.

For the purposes of this paper, it is important to note that only the health sector privacy laws in the provinces of New Brunswick[8], Newfoundland and Labrador[9], Nova Scotia[10] and Ontario[11] have been designated substantially similar to PIPEDA. That in the provinces of Alberta, Manitoba and

---

[7] At: https://www.parl.ca/legisinfo/en/bill/44-1/c-27.
[8] https://laws.gnb.ca/en/showfulldoc/cs/P-7.05/20121030
[9] https://assembly.nl.ca/Legislation/sr/statutes/p07-01.htm
[10] https://novascotia.ca/dhw/phia/PHIA-legislation.asp
[11] https://www.ontario.ca/laws/statute/04p03

Saskatchewan have not. The health information privacy legislation was enacted prior to the coming into force of PIPEDA and, based on the key "substantially similar" criterion of being consent-based, as is PIPEDA, would not have achieved this status even if enacted subsequently to the coming into force of PIPEDA.

The Public/Broader Public Sector

Each Canadian province and territory has its own public sector privacy legislation: it is generally combined with a right to access, subject to certain limited exceptions to information, including personal information in the custody or in the control of an enumerated list of public and broader public entities that are subject to the legislation.

Treatment and Definitions

Tables S1 (Private Sector), S2 (Health Information Specific) and S3 (Public Sector) of Appendix A set out the legislative definitions of personal or personal health information in each of the three sectors described above. For the purposes of this paper, the following points related to the definitions of personal information and personal health information should be kept in mind:

- *The Private Sector*: Prior to the coming into force of Quebec Bill 64, none of the Canadian private sector legislation provided any guidance on the interpretation of "identifiable" as is required for information about an individual to meet the definition of "personal information" and thus be subject to the legislation. As noted in Table S1, Bill 64 (which amended the Quebec private sector privacy law) introduced two substantive changes related to the definition of "personal information". The first was the definition itself which clarified that "de-identified information" is information that no longer allows a person to be **directly** identified". At first blush this appears to broaden the type of information that would be considered to fall outside of the application of the privacy law (i.e., because de-identified information could include that which could lead to the identification of an individual based on indirect or quasi-identifiers being included in the data. However, Bill 64 introduced the concept of "anonymization" to mean "information that at all times, reasonably foreseeable in the circumstances that it irreversibly no longer allows the person to be identified directly or indirectly". Accordingly, information that may have previously been considered to not be "personal information" because of the lack of reasonable foreseeability with respect to the inclusion of indirect identifiers, appears to clearly indicate that such information would be considered the protection to be personal information unless it was clear that there was no possible way of the information being re-identified. In

addition, subsequent to the conduct of the interviews with privacy regulators described in this paper, the Quebec government enacted its Final Anonymization Regulation.[12]

- Part I of the federal government Bill C-27 would enact the *Consumer Privacy Protection Act* (the CPPA) and repeal Part I of PIPEDA (the part dealing with the protection of personal information in the private sector). As is the case in Quebec Bill 64, Bill C-27 would also introduce the concept of "anonymize" in relation to personal information. "Anonymize" is defined as meaning to irreversibly and permanently modify personal information, in accordance with generally accept red best practices, to ensure that no individual can be identified from the information, whether directly or indirectly, by any means". This definition is arguably more stringent than that in Quebec Bill 64 in that it requires a degree of certainty (i.e., **ensure that no individual can be identified from the information**), rather than the lesser standard of it **not being reasonably foreseeable in the circumstances that it irreversibly no longer allows the person to be identified** …")

- The definition of "personal information" as meaning "information about an identifiable individual" remains the same as in PIPEDA; however, Bill C-27 includes a definition of "de-identify" meaning "to modify information so that an individual cannot be **directly identified from it, though a risk of an individual being identified remains**". Thus, the proposed federal scheme clearly articulates that if an individual may be indirectly identified from the data (regardless of whether the risk of identification is "reasonably foreseeable" or not), the information is still characterized as being "personal information". This is made explicitly clear in subsection 2(3) in which, subject to certain exceptions, interprets de-identified as being personal information.

- One of the exceptions – that in subsection 39(1) – disclosure for socially beneficial purposes – would further complicate the already complex Canadian privacy environment and has been the subject of much controversy (include submissions). This provision would permit an organization to disclose an individual's personal information without their knowledge or consent if a number of conditions were satisfied: i) the information is de-identified before the disclosure is made; ii) the disclosure is limited to a number of undefined entities, in the main described as institutions in Canada; or any that may be prescribed by regulation and iii) the disclosure is made for a

[12] At: https://www.publicationsduquebec.gouv.qc.ca/fileadmin/gazette/pdf_encrypte/lois_reglements/2024A/106829.pdf

"socially beneficial purpose" – a purpose " related to health, the provision or improvement of public amenities or infrastructure, the protection of the environment or any other prescribed purpose". Given that there is uncertainty with respect to both the entities to which organizations may disclose de-identified information (and the rationale for the listed inclusions) and the purposes for which they may do so, this provision has been the subject of much commentary.

- *The Health Sector:* The addition of terminology used in Quebec's Bill 64 and the proposed PIPEDA amendments – the definition of de-identified information, the inclusion of a new category of "anonymized information" and the uncertain restrictions on the disclosure of de-identified information in the federal Bill C-27 discussed above, further complicate the definitions noted in Table S2 – the health information specific Canadian privacy laws, especially that in Ontario – PHIPA – that has been determined to be substantially similar to PIPEDA. This results from the fact that, in addition to not including a definition of "anonymized information", PHIPA currently has a "placeholder" for a proposed, but not yet enacted, definition of "de-identify" that would amend the current definition to require the removal, **in accordance with such requirements as may be prescribed, any information …"** that identifies the individual or for which it is reasonably foreseeable in the circumstances that it could be utilized, either alone or with other information, to identify the individual …"
- This provision and those in the Northwest Territories *Health Information Act* and Regulations (set out in Table S2), do point to a path forward for the use of legislative/regulatory means to provide proactive objective direction to not only entities subject to the provisions of Canada's health information privacy laws, but also those in the private and public sectors as well. Section 85 in conjunction with section X of the Regulation, provide, albeit at this time at a reasonably high level, a potential way forward to establish legislative standards for the de-identification of personal and personal health information.
- *The Public Sector:* To date, the Canadian public sector privacy laws have fallen behind those in the private and health information sectors. Table S3 illustrates the consistency in the definition of "personal information" in these statutes, while at the same time highlighting the paucity of information available to assist both privacy regulators and institutions subject to these laws with direction and techniques to remove information that is not about an "identifiable" individual from the scope of application of these laws.
